# On the Removal of Warts

**Published:** 1882-02

**Authors:** 


### ON THE REMOVAL OF WARTS.

Dr. W. Allan Jamieson says, in the *Practitioner* for September, 1881, that chromic acid, one to one of water, is by far the best remedy. The skin round each wart is first proteced by painting it with oil, and then the wart itself is soaked with the solution of chromic acid; this absorbs water from the tissues, coagulating and hardening the albuminous tissues at the same time, and the unsightly warts soon disappear. These warts seldom appear after puberty on the hands. but a healthy girl well grown, aged fifteen, came to the writer sometime since with a dozen of them on her hands, which annoyed her for six years. Of course they much interfered with work, being always in the way. Steady use of the chromic acid removed them in a few weeks.

